# Bipolar Disorder VII: Family History and Its Relationship With Suicide Attempts, Severity, and the Prophylactic Effect of Lithium Treatment in a Long-Term Follow-Up Study of Bipolar Disorder

**DOI:** 10.1016/j.bpsgos.2025.100608

**Published:** 2025-09-06

**Authors:** Per-Olof Nylander, Erik Lexne, Christer Lehman, Lars Brudin, Finn Bengtsson

**Affiliations:** a Linköping University, Linköping, Sweden; b Academic Clinic of Färjestaden, Färjestaden, Sweden; c Forensic Psychiatric Regional Clinic, Sundsvall, Sweden; d Department of Medical and Health Science, Linköping University, Linköping, Sweden; e Department of Biomedical and Clinical Sciences, Linköping University, Linköping, Sweden

**Keywords:** Age of onset, Bipolar disorder, Episodes of disorder, Family history, Lithium drug therapy response, Long-term follow-up, Suicide attempts

## Abstract

**Background:**

Family history (FH) of affective disorders (ADs) is important for the course of bipolar disorder (BD).

**Methods:**

In a long-term study (mean 25 years), 192 patients with BD diagnosed by DSM-IV criteria were recruited from lithium dispensaries. Differences between patients with and without an FH of ADs were studied.

**Results:**

Patients with an FH of AD had poorer lithium response (*p* = .027), earlier age of onset (AOO) (*p* < .001), were younger (*p* = .009), made suicide attempts (SAs) earlier after onset (*p* = .012), and had more episodes/year (*p* = .017) and depressive episodes/year (*p* = .010) before SA. SAs were more common (*p* = .028) in patients with an FH of AD. SAs were more common (*p* = .001) before lithium treatment, and SAs (*p* < .001) were only present in patients with an FH of AD within the first 5 years after AOO. Patients with an FH of AD had more episodes (*p* = .009), episodes/year (*p* = .002), depressive episodes (*p* < .001), and depressive episodes/year (*p* < .001) during their lifetime. Before lithium, episodes (*p* = .009), depressive episodes (*p* = .006), and depressive episodes/year (*p* = .010) were more common in patients with an FH of AD. Manic episodes (*p* = .020) were more common in patients with no FH of AD. On lithium, episodes (*p* = .010), episodes/year (*p* = .001), depressive episodes (*p* < .001), and depressive episodes/year (*p* < .001) were more common in patients with an FH of AD. FH of suicide was present only among patients with an FH of AD (*p* < .001).

**Conclusions:**

BD patients with an FH of AD have a more severe form of BD with a special effect on SAs, AOO, episodes, and lithium response in BD.

## Plain Language Summary

Patients with a family history (FH) of affective disorders (ADs) had poorer lithium response, earlier age of onset (AOO), were younger, performed suicide attempts (SAs) earlier, had higher episodes/year and depressive episodes/year before SA. SAs were more common in patients, before lithium and only present 5 years after AOO. Patients had more episodes, episodes/year, depressive episodes, and depressive episodes/year during lifetime. Before lithium, episodes/year, depressive episodes, and depressive episodes/year were more common in patients. Manic episodes were more common in nonpatients. On lithium, episodes, episodes/year, depressive episodes, and depressive episodes/year were more common in patients. FH of suicide was only present among patients.

Bipolar disorder (BD) is a widespread common chronic affective disease, and approximately 1% of the general population is affected by the disorder. BD is characterized by recurrent depressive, hypomanic, and manic episodes. BD has a serious impact on reducing quality of life (1,2). Earlier studies (3, 4, 5, 6) of BD families, twins, and adoptees have shown a strong hereditable component in BD. Previous genome-wide association studies [for a review, see (7)] have identified and suggested several different gene loci associated with BD. The genetic background in BD on state of mood and heredity is still unclear.

Previous studies of twins and adoptees have shown the presence of heritable risk factors for suicidal behavior (3, 4, 5, 6, 7, 8, 9, 10, 11, 12, 13). Suicidal behavior, such as suicide and suicide attempts (SAs), are common in BD and are estimated to be approximately 10% to 15% (14,15) and 25% to 56% (14), respectively. Treatment with lithium as a mood stabilizer is well established in BD, and lithium prophylaxis is well known to effectively prevent SA and suicide in BD (16,17).

Previous studies have also shown that a family history (FH) of BD was associated with more suicidal ideation, SA (18), as well as completed suicide (19,20). Another previous study (21) found a higher lifetime prevalence of BD among first-degree relatives of probands who had died by suicide, and the prevalence of BD in families was associated with an increased risk of developing mood disorders and SA or suicide.

Moreover, other previous studies (22,23) of patients with BD found that SAs were related to an FH of affective disorders (ADs). An early SA within the first 5 years after age of onset (AOO) of BD was found to be associated with an FH of a first- and/or second-degree relative with AD (24). In a study (25) of young patients with early-onset BD, researchers found that an FH of depression was associated with SA. A higher risk for suicidal behavior in relatives of probands with psychiatric disorders in general and in mood disorders in particular have been found in several previous studies (26, 27, 28).

An FH of suicide has been shown to increase the suicide risk for patients with a major AD (29). Other earlier studies (30,31) have shown a higher risk for suicidal behavior in relatives of patients with SA or completed suicide. Another previous study (32) has shown that an FH of suicidal behavior increased the risk for major AD and suicidal behavior in first-degree relatives. Furthermore, yet another study (33) of patients with BD found that an FH of SA was the most important predictor of future suicidal behavior. It has also been suggested that an FH of suicidal behavior is a more important risk factor than an FH of AD for suicidal behavior in major ADs (34,35).

A previous study (36) has shown that an FH of affective illness or substance abuse among first-degree relatives was strongly associated with AOO in BD and in particular with childhood onset of the disease but declined with higher AOO. Several studies (37, 38, 39, 40, 41, 42) have found an earlier AOO in BD patients with an FH of AD. Furthermore, one study (43) found that more parental and grandparental psychiatric illness in general was associated with an earlier AOO of BD and was also strongly related to a poor long-term prognosis of the disease.

There are several previous studies of differences in episode frequencies between BD patients with and without an FH of AD (40,41,44, 45, 46). Those studies have shown that patients with BD belonging to the second generation of BD had a higher frequency of episodes compared with the first generation. Together, these studies suggest that the second generation of BD has a more severe form of the disorder. Another study also found a higher episode frequency among BD patients with an FH of AD compared with those without an FH of AD (47).

Patients with BD require long-term prophylactic treatment to prevent new episodes in the future. Lithium as a mood stabilizer is effective in preventing depressive and especially manic episodes in BD (48, 49, 50, 51, 52, 53, 54). Several previous studies have found that an FH of BD (38,55, 56, 57, 58) or AD (59,60) was more prominent in responders of lithium treatment compared with nonresponders. However, 2 other studies did not find any relationship between FH and lithium treatment response (61,62). An FH of unipolar affective disorder among relatives was not predictive of lithium response in patients with BD. One study (63) found that BD patients with an FH of mania or depression had a better response to lithium than those without an FH. Another study (64) found that responders to lithium treatment tended (not statistically significant) to have greater family loading for AD and alcoholism. However, they found no differences between responders and nonresponders on AOO, duration of illness, or episodes per year. Furthermore, 2 other studies (40,47) have found a correlation between a poorer lithium prophylactic effect and an FH of BD. A recent study (7) found that BD patients with a familial psychiatric history had more severe disease and a poorer treatment response.

In several previous studies, an FH of AD has (7,37,40,41,44, 45, 46, 47) been shown to cause increased severity of BD, for example in an earlier AOO, an increased number of episodes, hospitalizations, SA, and poorer lithium response.

Our hypothesis is also that BD patients with an FH of AD have a more severe form of BD. The aim of the current study was to look for differences between BD patients with and without an FH of AD and its impact on SA, severity of BD, and lithium treatment response. However, it is important with long-term observations to determine true differences in the clinical course of BD patients with and without an FH of AD investigated both before and on lithium treatment. For this purpose, a long-term 25-year follow-up study of 192 patients with BD sampled from lithium dispensaries was used in our study.

## Methods and Materials

A total of 192 patients with BD were recruited from the 3 lithium dispensaries of Umeå, Sundsvall, and Härnösand Hospitals in northern Sweden. Before assessing the data, we compared age, gender, and number of BD type I and II cases, respectively, from each dispensary. It was found that although the 3 dispensaries included a different number of patients (*n* = 106, 52, and 34, respectively), none of the parameters above were significantly different across investigation sites, thus allowing us to pool the patients into one cohort.

Patients with diagnoses of alcoholism, drug abuse, epilepsy, or mental retardation were excluded. For all included patients, medical records were available containing detailed information about childhood, social conditions, personal development, previous psychiatric illness, episodes, AOO, FH, and medical treatment. All patients were continuously followed from the date of onset of BD, and all episodes were documented by experienced psychiatrists. Furthermore, for all patients, BD diagnoses and all other data were scrutinized and confirmed by 2 of the authors (CL and P-ON, as experienced psychiatrists by training). This process also included interviews using the Present State Examination (PSE-10) (65) and checking all patients’ data by applying the Operational Criteria Checklist (OPCRIT) (66). The PSE-10 and OPCRIT were performed once by one of the authors (CL or P-ON) and used to confirm the diagnosis. Interrater reliability (IR) was 1.0 for diagnoses, episodes, AOO, and all other clinical variables. This very good precision in IR was probably based on the fact that CL and P-ON worked in parallel at the same clinic for many years. The process of sampling data was performed during the last 2 years of the project. The interviews were conducted when all data were sampled for the study at the end of the follow-up study (the last 2 years).

DSM-IV criteria (67) were used to diagnose BD type I and II. An affective episode was defined according to DSM-IV and Research Diagnostic Criteria for major depressive, hypomanic, or manic episode, requiring hospitalization or treatment as an outpatient. AOO was defined by the first episode of depression, hypomania, or mania. We also defined whether the first episode was a depressed, hypomanic, or manic episode. All episodes of depression, hypomania, and mania were plotted in a lifetime diagram from AOO to the end of the study, which made it possible to determine when each episode occurred. For each patient, information was obtained regarding the total number of episodes and frequency of depression, hypomania, and mania from AOO to the end of study. The number of years from AOO until criteria for diagnoses of BD were met was recorded.

Data concerning lithium treatment were divided into length of time before lithium treatment was introduced and when on/off lithium therapy occurred. This made it possible to measure episode frequency before initiation of lithium and the effect of on/off lithium dispensation. Treatment response was calculated by comparing the number of episodes per year while off and on lithium.

Compliance was defined by measurement of serum lithium (S-lithium) levels (blood samples) and medical records. Poor compliance was defined when notes in the medical record reported discontinuation of medication or if S-lithium levels were inadequately low or were fluctuating over time. The latter was defined if S-lithium displayed a significant variation despite an unchanged dose of lithium or if a change in lithium dose was not followed by an expected change in the concentration of S-lithium. The usual therapeutic interval for S-lithium for most patients is between 0.5 and 0.9 mmol/L, referring to trough levels (i.e., sample drawn before next dose) at steady state (usually achieved after 1 week of daily intake of the drug). The treatment goal is to have the optimal effect at as low a dose as possible. S-lithium levels >1.2 mmol/L are considered to increase the risk for intoxication and >3.0 mmol/L to be life-threatening and potentially lethal.

SA at first episode from AOO was defined as well as SA before or after initiation of lithium treatment. The number of years to the first SA was defined as the number of years from the AOO. SAs were defined in the same way within the first 0 to 5 years, 0 to 10 years, 0 to 15 years, 0 to 20 years, and 0 to 25 years from the AOO. Whether patients had single or multiple SAs was also tracked.

FH information was obtained in detail from medical records and by semistructured interviews of patients and relatives by 2 of the authors (CL and P-ON) and by using the Family History Research Diagnostic Criteria (68) for definition of FH of AD (major depression, recurrent depression, BD type I and II) and FH of suicide. Only patients with an FH of AD among first- and/or second-degree relatives were defined as having an FH of AD. Altogether, we found 418 first- (*n* = 242) or second-degree (*n* = 176) relatives with an AD. Patients with an FH of psychiatric disorders other than AD were not included in this study to avoid a heterogenic background. Patients were divided into 2 groups, those with or without an FH of AD.

Before initiation of lithium treatment, a depressive episode was treated with antidepressants and, if needed, in combination with hypnotics and anxiolytics. For all patients, medication was registered in medical records at the psychiatric clinic. When lithium treatment started, lithium treatment was used alone and not in combination with any other psychotropic drug, except for hypnotics and anxiolytics when clinically indicated.

Statistics were calculated with SPSS version 23 (IBM Corp.) and Statistica StatSoft, Inc. version 12 (only for Kaplan-Meier analyses). χ^2^ with Fisher’s exact test (2 tailed) was used for analysis of prevalence based on categorical variables. For comparisons of continuous variables, Student’s *t* test (normal distribution) and the Mann-Whitney *U* test (non-normal distribution) were used. The significance level chosen was *p* < .05. The possibility of the occurrence of multiple significance errors was considered. However, because the parameters obtained in most cases were regarded as very different and most likely unrelated, we decided not to correct the statistical analysis for the risk of multiple erroneous significant findings. Such interventions, such as using the Bonferroni correction algorithm for example, may themselves risk underestimating existing significant differences and thereby risk introducing yet another bias instead.

The Ethical Committee in Umeå (Sweden) approved the study (Dnr. 92-045, owned by the author P-ON).

## Results

Information on gender, diagnoses, first episode depression, treatment response, compliance, FH of suicide, age, AOO, first episode of depression/hypomania/mania, and time to reach a bipolar diagnosis are shown in Table 1. BD patients with an FH of AD had a significantly (*p* = .027) poorer treatment response on lithium compared with BD patients without an FH of AD. An FH of suicide was significantly (*p* < .001) more common in patients with an FH of AD and was also only present in patients with an FH of AD. AOO was significantly (*p* < .001) earlier in BD patients with an FH of AD, and they were also significantly (*p* = .009) younger compared with BD patients without an FH of AD.

**Table 1:** Demographic and Clinical Characteristics of 192 BD Patients With and Without a Family History of ADs

	Family History of AD		*p*
	No, *n* = 54	Yes, *n* = 138	
Gender, Female	28 (51.9%)	84 (60.9%)	n.s.
Diagnosis			
BD I	46 (85.2%)	109 (79.0%)	n.s.
BD II	8 (14.8%)	29 (21.0%)	
First Episode Depression	34 (63.0%)	100 (72.5%)	n.s.
Treatment Response, Complete	16 (29.6%)	21 (15.2%)	.027
Compliance, Poor	24 (44.4%)	65 (47.1%)	n.s.
Family History of Suicide	0 (0.0%)	41 (29.7%)	<.001
Age, Years	61.74 ± 13.12	55.29 ± 15.30	.009
AOO, Years	36.09 ± 11.29	28.79 ± 10.03	<.001
First Depression, Years	1.47 ± 3.69	0.97 ± 3.49	n.s.
Bipolar Diagnosis, Years	9.05 ± 12.20	7.30 ± 9.03	n.s.
First Episode Depression, Years	1.67 ± 1.33	1.51 ± 1.27	n.s.
First Episode Hypomania, Years	3.95 ± 4.16	4.44 ± 4.14	n.s.
First Episode Mania, Years	2.86 ± 2.18	4.41 ± 4.70	n.s.

Values are presented as *n* (%) or mean ± SD. AD indicates ADs among first- and second-degree relatives. Complete treatment response was calculated by comparing number of episodes/year off and on lithium. Poor compliance indicates that the participants showed signs of not taking medicine as prescribed. Bipolar diagnosis indicates years until the participant fulfilled the criteria for BD.

AD, affective disorder; AOO, age of onset; BD, bipolar disorder; n.s., not significant.

Table 2 shows SAs during a long-term follow-up in BD patients with and without an FH of AD. In general, SAs were significantly (*p* = .028) more common among patients with an FH of AD compared with patients without an FH of AD. During the first 5 years after AOO, only patients with an FH of AD made an SA (*p* < .001). In all periods after the first 5 years after AOO, SAs were significantly more common in patients with an FH of AD. SAs were also significantly (*p* = .001) more common among patients with an FH of AD before they were introduced to lithium treatment.

**Table 2:** SAs in 192 BD Disorder Patients With and Without a Family History of ADs

	Family History of AD		*p*
	No, *n* = 54	Yes, *n* = 138	
SA	8 (14.8%)	43 (31.2%)	.028
SA at AOO	0 (0.0%)	10 (7.2%)	n.s.
SA First 5 Years	0 (0.0%)	24 (17.4%)	<.001
SA First 10 Years	3 (5.6%)	30 (21.7%)	.006
SA First 15 Years	5 (9.3%)	33 (23.9%)	.026
SA First 20 Years	5 (9.3%)	35 (25.4%)	.017
SA First 25 Years	7 (13.0%)	38 (27.5%)	.037
SA Before Lithium	3 (5.6%)	35 (25.4%)	.001
SA on Lithium	1 (1.9%)	9 (6.5%)	n.s.
Multiple SAs	2 (3.7%)	17 (12.3%)	n.s.

Values are presented as *n* (%). AD indicates ADs among first- and second-degree relatives.

AD, affective disorder; AOO, age of onset; n.s., nonsignificant; SA, suicide attempt.

Years, episodes, and episodes per year before SA in BD patients with and without an FH of AD are shown in Table 3. Patients with an FH of AD made SAs significantly earlier (*p* = .012) than patients without an FH of AD. Both episodes per year (*p* = .017) and depressive episodes per year (*p* = .010) before SA were significantly higher in patients with an FH of AD compared with patients without an FH of AD.

**Table 3:** Years, Episodes, and Episodes per Year Before SAs in 192 BD Patients With and Without a Family History of ADs

	Family History of AD		*p*
	No, *n* = 54	Yes, *n* = 138	
Years Before SA	16.56 ± 9.87	8.77 ± 11.24	.012
Episodes Before SA	8.38 ± 4.47	6.63 ± 6.96	n.s.
Episodes/Year Before SA	0.65 ± 0.42	1.37 ± 0.97	.017
Depressive Episodes Before SA	5.25 ± 4.65	4.47 ± 4.65	n.s.
Depressive Episodes/Year Before SA	0.37 ± 0.25	1.14 ± 0.96	.010

Values are presented as mean ± SD. AD indicates ADs among first- and second-degree relatives. Years, episodes, and episodes/year indicate number of years, episodes, and episodes/year from AOO until the first SA.

AD, affective disorder; AOO, age of onset; n.s., not significant; SA, suicide attempt.

Total years, episodes, and episodes per year in BD patients with and without an FH of AD are shown in Table 4. Patients with an FH of AD had significantly more episodes (*p* = .009) and episodes per year (*p* = .002) compared with patients without an FH of AD. Furthermore, patients with an FH of AD had significantly more depressive episodes (*p* < .001) and depressive episodes per year (*p* < .001).

**Table 4:** Total Years, Episodes, and Episodes per Year in 192 BD Patients With and Without a Family History of ADs

	Family History of AD		*p*
	No, *n* = 54	Yes, *n* = 138	
Years	24.72 ± 11.21	25.26 ± 12.86	n.s.
Episodes	11.31 ± 6.34	14.90 ± 8.60	.009
Episodes/Year	0.51 ± 0.31	0.70 ± 0.40	.002
Depressive Episodes	5.37 ± 5.11	8.92 ± 6.51	<.001
Depressive Episodes/Year	0.23 ± 0.22	0.40 ± 0.26	<.001
Hypomanic Episodes	2.37 ± 2.45	3.27 ± 4.19	n.s.
Hypomanic Episodes/Year	0.11 ± 0.14	0.15 ± 0.17	n.s.
Manic Episodes	3.57 ± 3.11	2.83 ± 2.83	n.s.
Manic Episodes/Year	0.18 ± 0.19	0.16 ± 0.20	n.s.

Values are presented as mean ± SD. AD indicates ADs among first- and second-degree relatives. Years, episodes, and episodes/year indicate number of years, episodes, and episodes/year from age of onset until the end of the study.

AD, affective disorder; n.s., not significant.

Table 5 shows years, episodes, and episodes per year before initiation of lithium treatment and while on lithium in BD patients with and without an FH of AD. Before lithium, the total episodes was significantly (*p* = .009) more common in patients with an FH of AD. Furthermore, patients with an FH of AD had significantly more depressive episodes (*p* = .006) and depressive episodes per year (*p* = .010). Manic episodes per year were significantly more common in patients without an FH of AD.

**Table 5:** Years, Episodes, Episodes per Year Before and While on Lithium Treatment, and S-Lithium Values in 192 BD Patients With and Without a Family History of ADs

	Family History of AD		*p*
	No, *n* = 54	Yes, *n* = 138	
Before Lithium			
Years	9.17 ± 9.45	12.32 ± 11.57	n.s.
Episodes	5.74 ± 3.96	8.22 ± 7.21	.009
Episodes/year	1.33 ± 1.16	1.30 ± 1.26	n.s.
Depressive episodes	3.33 ± 3.48	5.28 ± 5.96	.006
Depressive episodes/year	0.58 ± 0.68	0.67 ± 0.60	.010
Hypomanic episodes	0.85 ± 1.41	1.42 ± 2.58	n.s.
Hypomanic episodes/year	0.17 ± 0.35	0.21 ± 0.39	n.s.
Manic episodes	1.56 ± 1.30	1.51 ± 1.69	n.s.
Manic episodes/year	0.59 ± 0.71	0.41 ± 0.85	.020
On Lithium			
Years	13.51 ± 7.81	12.25 ± 8.34	n.s.
Episodes	3.09 ± 3.36	5.16 ± 5.40	.010
Episodes/year	0.25 ± 0.26	0.50 ± 0.48	.001
Depressive episodes	1.07 ± 1.34	2.91 ± 3.50	<.001
Depressive episodes/year	0.10 ± 0.13	0.28 ± 0.33	<.001
Hypomanic episodes	1.09 ± 1.58	1.36 ± 2.20	n.s.
Hypomanic episodes/year	0.08 ± 0.13	0.12 ± 0.18	n.s.
Manic episodes	0.93 ± 1.65	0.88 ± 1.50	n.s.
Manic episodes/year	0.08 ± 0.16	0.10 ± 0.19	n.s.
S-lithium, no episodes, mmol/L	0.61 ± 0.12	0.61 ± 0.11	n.s.
S-lithium, episodes, mmol/L	0.59 ± 0.14	0.61 ± 0.12	n.s.

Values are presented as mean ± SD. AD indicates ADs among first- and second-degree relatives. Years, episodes, and episodes/year indicate number of years, episodes, and episodes/year from AOO until the end of the study.

AD, affective disorder; AOO, age of onset; n.s., not significant; S-lithium, serum lithium.

On lithium, episodes (*p* = .010) and episodes per year (*p* = .001) were significantly more common in patients with an FH of AD. The total number of episodes decreased by 46.2% in patients without an FH of AD and by 37.2% in patients with an FH of AD. Depressive episodes (*p* < .001) and depressive episodes per year (*p* < .001) were also significantly more common while on lithium in patients with an FH of AD. The total number of depressive episodes decreased by 67.9% in patients without an FH of AD and by 44.9% in patients with an FH of AD. The total number of depressive episodes per year decreased by 82.8% in patients without an FH of AD and by 58.2% in patients with an FH of AD.

**Figure 1:**
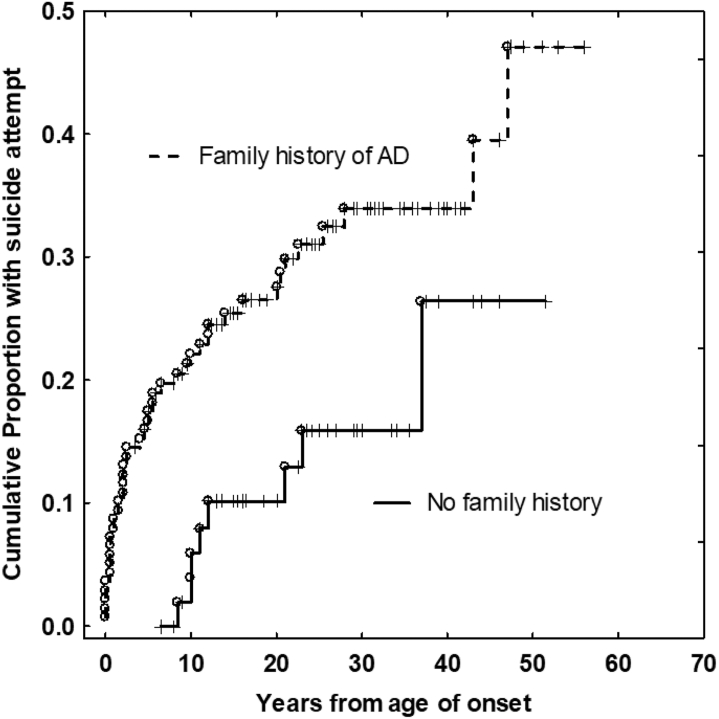
Kaplan-Meier diagram shows the cumulative proportion of patients with suicide attempts for those with family history (FH) of affective disorders (ADs) (among first- and second-degree relatives) (upper curve) compared with those with no FH (lower curve). The difference is significant at *p* = .005 (log-rank test).

Differences in SA between patients with and without an FH of AD were statistically significant (*p* = .005) and were assessed using the Kaplan-Meier estimator (Figure 1).

## Discussion

An FH of AD or suicide is obviously important for the course of BD. These factors seem to have an impact on several aspects of BD, including for example suicidal behavior, AOO (36, 37, 38, 39, 40, 41, 42, 43), and the severity of the disease (18, 19, 20, 21, 22, 23, 24, 25, 26, 27, 28, 29, 30, 31, 32, 33).

In clinical practice, a correct FH in all details is often very hard to obtain from patients. Thus, a great variety of psychiatric disorders of relatives is often reported by different patients upon questioning by the physician. This fact is taken into consideration in the current investigation in that we considered the current various affective conditions together in our definition of AD. This is also the way that previous studies have commonly handled the notion of AD (7,22, 23, 24, 25, 26, 27, 28,36, 37, 38, 39, 40, 41, 42, 43, 44, 45, 46, 47,59, 60, 61, 62, 63, 64).

Several previous studies have found that an FH of AD (18, 19, 20, 21, 22, 23, 24, 25, 26, 27, 28) and suicidal behavior (29, 30, 31, 32, 33) increased the presence for suicidal behavior among relatives. We also found that SAs were significantly more common among BD patients with an FH of AD, and SAs were twice as common in patients with an FH of AD compared with patients without an FH of AD. Only BD patients with an FH of AD made SAs during the first 5 years after AOO. The same group of patients predominated when SA occurred during the first 25 years after AOO. We emphasize the importance of penetrating an FH of AD because this is obviously associated with a higher risk that an SA has been or will be made in relatives with BD.

Overall, BD patients with an FH of AD had an earlier first SA (approximately 8 years) compared with BD patients without an FH of AD. Patients with an FH of AD had an equal number of episodes before the first SA compared with patients without an FH of AD but during a much shorter period of time. Therefore, during this period, they had significantly more episodes per year before performing their first SA, and this was mainly due to significantly more depressive episodes per year.

It has previously been suggested that an FH of suicidal behavior is an even more important risk factor for SA than an FH of AD (34,35). However, suicidal behavior is very common in patients with AD in general (14,15). Therefore, as expected, an FH of suicide was significantly more common in patients with an FH of AD. We also found that an FH of suicide was only present among patients with an FH of AD.

If an FH of suicide is independent of an FH with or without AD, then an FH of suicide should be approximately equally distributed in both groups and not especially connected with an FH of AD. According to our results, an FH of suicide and an FH of AD should be regarded as the same type of FH. Our data suggest that it is important to determine whether a patient with BD has an FH of AD and that an FH of AD is most important because it is associated with a higher risk for suicidal behavior in relatives with BD.

We found that BD patients with an FH of AD were significantly younger and that AOO was significantly earlier (approximately 7 years) among BD patients with an FH of AD. Furthermore, we found that overall, BD patients with an FH of AD had significantly more episodes and episodes per year compared with BD patients without an FH of AD. This was mainly due to significantly more depressive episodes and depressive episodes per year.

Furthermore, BD patients with an FH of AD had more depressive episodes and fewer manic episodes before initiation of lithium treatment, and their first manic episode generally appeared later after the AOO than in BD patients without an FH of AD. Therefore, BD patients with an FH of AD may be misdiagnosed, regarded, and treated as having unipolar affective disorder for longer at the beginning of their illness. This may be an explanation for why those patients are not treated with mood stabilizers earlier in the course of the disease compared with BD patients without an FH of AD. Probably as a consequence of this, BD patients with an FH of AD made an SA significantly more often before initiation of lithium treatment compared with BD patients without an FH of AD. Another explanation for more SAs before initiation of lithium treatment among BD patients with an FH of AD is that they generally make their first SA much earlier compared with those without an FH of AD.

We found that the number of episodes per year decreased with lithium treatment in both BD patients with and without an FH of AD. In summary the number of overall episodes (62.4% vs. 46.9%) and episodes per year (78.3% vs. 59.7%) decreased more in BD patients without an FH of AD compared with patients with an FH of AD. BD patients with an FH of AD had both significantly more episodes and more episodes per year while on lithium treatment. There was also a significant difference between the 2 groups on depressive episodes and depressive episodes per year on lithium, with the poorest lithium response among BD patients with an FH of AD. Furthermore, complete treatment response was significantly higher in patients without an FH of AD compared with patients with an FH of AD (approximately 30% vs. 15%). Altogether, our results shows that BD patients with an FH of AD have a poorer lithium response compared with BD patients without an FH of AD.

It has previously been pointed out that long-term observations both before and while on lithium treatment, as well as large number of patients, are necessary to determine differences in the clinical course between responders and nonresponders (55). Previous studies of lithium response (38,55, 56, 57, 58, 59,61, 62, 63) have mainly been conducted to determine whether there are any breakthrough episodes during a short period of usually 1 to 3 years. A short follow-up period has the advantage of better monitoring and the possibility of adjustment of lithium therapy when needed to avoid a new episode. On the other hand, a long follow-up period has the advantage of better estimation of relapse frequency per year both before and while on lithium treatment. To our knowledge, there have been few long-term follow-up studies of lithium response, both studies comparing episodes per year before initiation of lithium and while on lithium (47,60,69). Of these studies, the first one (47) found a poorer lithium response among patients with an FH of AD, the second one (60) found a correlation between an FH of BD and a better lithium response, and the third one found no better lithium response among patients with an FH of AD (69). In our study, the mean follow-up period was approximately 9 to 12 years before initiation of lithium therapy and 12 to 13 years on lithium therapy. S-lithium concentration was monitored according to the frequent routine of the lithium dispensaries, and consequently S-lithium values could be determined 3 months prior to relapses. Therefore, we can exclude the possibility that differences in S-lithium are an explanation for differences in episode frequency between patients with and without an FH of AD.

In several previous studies, responders have been defined as having no episodes at all during lithium therapy (55,56,58,59). This means that not all patients who respond positively, but only show partial improvement on lithium treatment, will be included in subsequent analyses. This method of characterizing responders (complete) will depend on the time elapsed during the follow-up period, which differs significantly between these studies and the current study. Therefore, the data in previous studies and the current study are not comparable. In our study, we have instead measured lithium response in percentage by comparing episodes per year before and while on lithium treatment.

It is well known that schizoaffective disorder or BD patients with schizophrenia in the family background have a poorer lithium response (55,70, 71, 72, 73, 74). In our study, we only included BD patients with an FH of AD and nonfamilial patients to avoid a heterogeneous background. Patients who are rapid cyclers are also known to have a poorer lithium response (75, 76, 77). Only 6 patients in our study were rapid cyclers (3.1%), 1 nonfamilial (1.9%) and 5 familial (3.6%), and therefore this did not interfere with the main results of the study.

Our result of a poorer lithium response among BD patients with an FH of AD is in good agreement with a few other studies (40,47). However, our results are the opposite of several other studies that found that BD patients with an FH of AD had a better response to lithium (38,55, 56, 57, 58, 59,61, 62, 63). This may be explained by the different inclusion criteria used as well as how responders were characterized in the previous studies in contrast to the current study. We also had a much longer follow-up period, both before and while on lithium therapy, which made it possible to better calculate lithium response by comparing episodes per year before and while on lithium treatment.

The size of the patient group that we studied may be considered not large enough to allow for overall generalizability of our results. However, we are not aware of any other long-term studies extending over 25 years of follow-up in patients with BD.

### Conclusions

To summarize, our results show that BD patients with an FH of AD have a more severe form of BD compared with BD patients without an FH of AD. An FH of AD in BD is linked to a higher frequency of SAs, an earlier AOO, a higher number of disease episodes, a higher frequency of episodes per year and in particular a higher number of overall depressive episodes and a higher frequency of depressive episodes per year as well as a poorer lithium response. Taken together, our results provides information suggesting that an FH of AD has a profound effect on the severity in the progress of a BD.

In our study, an FH of suicide was only found in patients with BD who also had an FH of AD. Therefore, we suggest that it is important for clinicians to investigate the possible presence of an FH of AD in parallel with an FH of suicide in patients with BD. The main clinical implications of the current study are to highlight the importance of detailed knowledge on the FH of AD among patients with a BD. This understanding will provide a possibility of better risk management of SAs as well as improvements in drug treatment with lithium. However, because of the current study’s retrospective design, we also encourage investigators to conduct further studies in the future, possibly with a prospective design, hopefully to confirm our results.

## Supplementary Material

Key Resources Table
